# Highly active antiretroviral therapy-silver nanoparticle conjugate interacts with neuronal and glial cells and alleviates anxiety-like behaviour in streptozotocin-induced diabetic rats

**DOI:** 10.1016/j.ibneur.2022.06.003

**Published:** 2022-06-16

**Authors:** Sodiq Kolawole Lawal, Samuel Oluwaseun Olojede, Ayobami Dare, Oluwaseun Samuel Faborode, Sheu Oluwadare Sulaiman, Edwin Coleridge Naidu, Carmen Olivia Rennie, Onyemaechi Okpara Azu

**Affiliations:** a Discipline of Clinical Anatomy, School of Laboratory Medicine & Medical Sciences, Nelson R Mandela School of Medicine, University of KwaZulu-Natal, 719 Umbilo Road, Durban, South Africa; b Department of Physiology, School of Laboratory Medicine & Medical Sciences, Westville Campus, University of KwaZulu-Natal, Durban, South Africa; c Department of Human, Biological & Translational Medical Sciences, School of Medicine, University of Namibia, Hage Geingob Campus, Private, Bag, 13301, Namibia; d Graduate Program in Cell Biology, Institute of Biological Sciences, Federal University of Minas Gerais (UFMG), Belo Horizonte, Brazil

**Keywords:** HAART, Prefrontal cortex, Glial fibrillary acidic protein, Silver nanoparticles, Inflammation, And oxidative stress

## Abstract

The inception of highly active antiretroviral therapy (HAART) has changed the management of human immunodeficiency virus (HIV) positive patients, with an improvement in life expectancy. However, neurological complications associated with high dosage and chronic administration of HAART have not been fully addressed. Therefore, this study evaluated the potential benefits of silver nanoparticles (AgNPs) conjugated-HAART (HAART-AgNPs) and its interaction with neuronal and glial cells in type-2 diabetic rats. Forty-two (n = 42) adult male Sprague-Dawley rats (250 ± 13 g) were divided into non-diabetic and diabetic groups. Each rat was administered with either distilled water, HAART, or HAART-AgNPs for eight weeks. After that, the prefrontal cortex (PFC) was excised for immunohistochemical, biochemical, and ultrastructural analysis. The formulated HAART-AgNPs were characterised by Ultraviolet-Visible, Transmission electron microscope, Energy Dispersive X-ray and Fourier transform infrared spectroscopy. Of the various concentrations of HAART-AgNPs, 1.5 M exhibited 20.3 nm in size and a spherical shape was used for this study. Administration of HAART-AgNPs to diabetic rats significantly decreased (*p < 0.05)* blood glucose level, number of faecal pellets, malondialdehyde (MDA), tumour necrosis factor-alpha (TNF-α)*,* Interleukin-1 beta (IL-1β) compared with HAART-treated diabetic rats. Notably, there was a significant increase (*p < 0.05)* in antioxidant biomarkers (SOD and GSH), improvement in PFC-glial fibrillary acid protein (PFC-GFAP) positive cells and alleviation of anxiety-like behaviour in HAART-AgNPs treated diabetic rats. These results showed that HAART-AgNPs alleviates the anxiogenic effect and neuronal toxicity aggravated by HAART exposure via the reduction of oxidative and neuroinflammatory injury as well as preserving PFC GFAP-positive cells and neuronal cytoarchitecture.

## 1 Introduction

The introduction of highly active antiretroviral therapy (HAART) has changed the Human Immunodeficiency Virus (HIV) diagnosis from a fatal disease into a chronically managed condition. Consequently, the life expectancy and the quality of life among the people living with HIV have since improved (Barbier et al., 2020). Despite the benefits of HAART, its long-term use and systemic exposure has been strongly linked with various metabolic disturbances such as diabetes and cardiovascular diseases (Nansseu et al., 2018, Sapula et al., 2022). Previous studies have reported that initiation of HAART increases the risk of diabetes mellitus (Nansseu et al., 2018, Ergin et al., 2020). The mechanism by which HAART causes diabetes has been attributed to excessive reactive oxygen species (ROS) production, leading to cell toxicity (Awodele et al., 2018, Ikekpeazu et al., 2020). The increased ROS damage vital cellular components (e.g., DNA, lipids, and proteins) in glucoregulatory tissues leading to insulin resistance or compromised insulin synthesis, thereby promoting hyperglycemia (Han, 2016). Also, HAART has been linked with mitochondrial damage and subsequently increases the risk of neuropathy and neuroinflammation (Lin et al., 2018, Lu et al., 2021). Chronic administration of HAART to HIV positive patients has been reported to cause neuroinflammation, changes in astrocyte mitochondrial membrane and mitochondrial ROS production in animal experiments (Bertrand et al., 2021, Lawal et al., 2022).

The astrocytes are the major components of the brain tissue involved in the overall maintenance of brain homeostasis, neuronal metabolism, and neuroprotection (Siracusa et al., 2019). Thus, active astrocyte dysfunction during hyperglycemia characterised by a decrease in glial fibrillary acidic protein (GFAP) level has been reported to promote neurocognitive dysfunctions (Yang et al., 2018, Kodidela et al., 2020).

The continuous use of HAART to prevent a viral rebound in people living with HIV and diabetes-induced neuroinflammation caused detrimental effects on astrocytes in the CNS and contributed significantly to the aetiology of neuro-pathologies (Cohen et al., 2017, Yang et al., 2018). Excessive production of pro-inflammatory cytokines during neuroinflammation has been implicated in cognitive deficits and anxiety disorders (Charlton et al., 2018, Li et al., 2019). Interestingly, in the post-era of HAART, people living with HIV have experienced an improvement in motor skills and verbal fluency but show impaired executive functions and anxiety-like behaviour (Heaton et al., 2011, Checa et al., 2020). In addition, the prevalence of anxiety and depression among the patient receiving HAART remains high (Nuesch et al., 2009, Rabkin et al., 2000). The most used components of HAART (Efavirenz and Tenofovir) have been reported to cross the blood-brain barrier, causing mitochondrial dysfunction and some neurological-related adverse effects like depression and anxiety disorder (Chen et al., 2019, Checa et al., 2020). Several studies have suggested that the prefrontal cortex and its circuitry play a vital role in anxiety-like behaviour in animals and humans (Likhtik et al., 2014, Hare and Duman, 2020). More so, a decrease in the prefrontal cortex activities and abnormalities in the neuroimaging studies have been observed in fearful and anxious individuals (Berkowitz et al., 2007, Likhtik et al., 2014).

The application of nanomedicine for antiretroviral drug delivery holds promise in HIV therapeutics due to their unique advantages such as increased drug bioavailability, stability, ability to reach the target cell population, and half-life (Kumar et al., 2015).

The primary issue with HAART is that it requires high doses for a prolonged duration of time to reduce the viral level in the system, thus predisposing living tissue to toxicity (Kumar, 2019).

Silver nanoparticles (AgNPs) exhibit novel properties, making them suitable for a wide range of applications in the biomedical field. In addition, AgNPs are the most studied and utilised nanoparticles due to their simple method of synthesis, high surface to volume ratio, unique morphology, and intracellular delivery system (Marin et al., 2015). AgNPs have been utilised as antiviral, antidiabetic, and antioxidant agents in the biomedical field (Vadlapudi and Amanchy, 2017).

Conversely, in vitro and in vivo studies on the neurotoxic effects of silver and silver nanoparticles reported a size- and dose-dependent cellular uptake and toxicity (Greish et al., 2019a, Ferdous and Nemmar, 2020). Small-medium-sized nanoparticles have been reported to be less toxic to the cell (Lara et al., 2010, Ferdous and Nemmar, 2020). More so, studies have reported that cytotoxic effects of silver nanoparticles can be minimised by reducing silver ions to a ground state (from Ag^+^ to Ag^0^), synthesising a spherical shape, small-medium size, and modified surface area (Smith et al., 2018, Dlugosz et al., 2020). Another study suggests that the cytotoxic effect observed in the use of silver nanoparticles is due to silver ions exposure (Wang et al., 2014).

However, there is no data to substantiate the interaction of HAART conjugated with silver nanoparticles on neuronal cells and neurocognitive dysfunctions. Hence, this study assessed the role of HAART-silver nanoparticles conjugate on the PFC of STZ-induced diabetic rats.

## 2 Materials and methods

### 2.1 Materials

The Atripla, a combined form of Efavirenz (EFV, 600 mg), Emtricitabine (FTC, 200 mg) and Tenofovir disoproxil fumarate (TDF, 300 mg), was purchased from Dis-Chem pharmacy Ballito, South Africa. Streptozotocin (STZ), trisodium citrate, Sodium hydroxide and silver nitrate (AgN0_3_) of analytical grade were sourced from Sigma-Aldrich Company, Johannesburg, South Africa. Enzyme-linked immunoassay (ELISA) kits for TNF-α (Catalogue no: E-EL-R0019) and interleukin (IL)− 1β (Catalogue no: E-EL-R0012) were purchased from BIOCOM Africa (pty), Ltd, South Africa. All the chemicals, reagents, and equipment were of analytical grade.

### 2.2 Experimental animal

Forty-two (42) adult male Sprague-Dawley rats (250 ± 13 g) were obtained from the Biomedical Research Unit (BRU) of the University of KwaZulu-Natal and were housed in the standard animal laboratory room. The animal laboratory room was maintained at a temperature of 24–26 ^°^C, 12:12 light: dark cycle and 40–60% humidity. The animals were allowed free access to water and feed ad libitum. All animals were handled according to the National Institute of Health Guide for the Care, and Use of Laboratory Animals (NIH Publications No. 80–23), revised in 1996. The animal laboratory procedures were approved by the Animal Ethics Committee of the University of KwaZulu-Natal (AREC/044/019D).

### 2.3 Experimental design

**Fig. 1:**
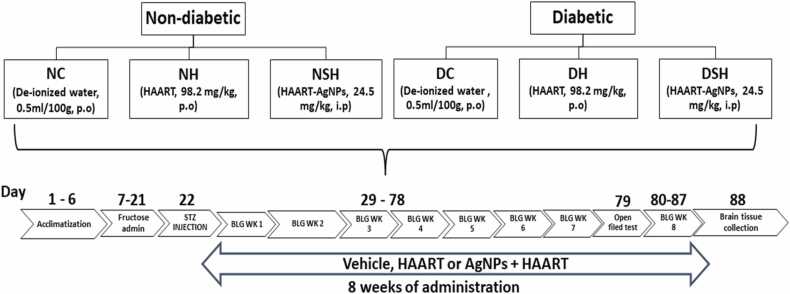
Illustrate experimental design. Group 1–3, designated as NC, NH and NSH were non-diabetic animals, but treated with vehicle (distilled water, 0.5 mL/100 g, p.o), HAART (98.2 mg/kg, p.o), and HAART-AgNPs (24.5 mg/kg, i.p). Group 4–6, designated as DC, DH and DSH were diabetic animals treated with (distilled water, 0.5 mL/100 g, p. o), HAART (98.2 mg/kg, p. o), and HAART-AgNPs (24.5 mg/kg, i.p). All rats were treated daily except for i.p groups, which were treated for 5 days per week for eight weeks. NC= non-diabetic control, NH= non-diabetic + HAART, NSH= non-diabetic + HAART-AgNPs, DC= diabetic Control, DH= diabetic + HAART, DSH= diabetic + HAART-AgNPs, i. p = intraperitoneal injection. p.o = per os, BLG WK= blood glucose weekly measurement, STZ= streptozotocin.

After acclimatisation for six (6) days, the rats were randomly divided into six groups (n = 7 per group) and were treated for eight weeks, as in Fig. 1. The recommended animal dose was calculated using a human equivalent dose (HED) as recommended by the United States Food and Drug Administration (FDA) (Nair and Jacob, 2016) and the dose given was determined according to the previous studies (Everson et al., 2018, Olojede et al., 2022).

### 2.4 Induction of type II diabetes in rats

Experimental type 2 diabetes mellitus was induced using a fructose-streptozotocin (STZ) rat model as described by (Wilson and Islam, 2012). Briefly, rats received 10% fructose solution ad libitum for two weeks. After that, the rats were fasted overnight and injected with a single of 40 mg/Kg B.W. STZ i.p. The STZ was dissolved in 0.9% NaCl with 100 mM sodium citrate buffer (pH 4.5). The control rats received an equal volume of the buffer. Animals with fasting blood glucose levels ≥ 200 mg/dL were considered diabetic and included in this study.

### 2.5 Formulation of HAART-AgNPs

Silver nanoparticles were synthesised according to Turkevich et al. (Turkevich et al., 1951). Briefly, an aqueous solution (0.03 M) of silver nitrate (AgNO_3_) was prepared from 5.10 g of AgNO_3_ crystal. Then, a stock aqueous solution (2 M) of trisodium citrate (TSC) was prepared from 147 g in 250 mL of double-distilled water and used as a reducing and stabilising agent. Four TSC solutions with varying concentrations (0.5 M, 1 M, 1.5 M & 2 M) were prepared. Thereafter, an aqueous solution of 0.03 M of AgNO_3_ was mixed with various concentrations of TSC, and it was continuously stirred for 5 mins at 90 ◦C. The resultant solution was adjusted with concentrated NaOH at a pH of 10.5. A colour change from colourless to amber yellow was observed, and this indicated that AgNPs was successfully synthesised.

The HAART-silver nanoparticle (HAART-AgNPs) was prepared by dissolving 15 g of HAART in 10 mL of concentrated sodium hydroxide solution, and distilled water was added to make 50 mL. The final concentration of HAART (1. 05 M) was mixed with 100 mL of AgNPs aqueous solution and then stirred on ultra-sonication to ensure proper reaction of HAART and AgNPs.

The conjugated AgNPs + HAART was centrifuged at 4500 rpm and 40 °C for 40 min to discrete the unincorporated drug. The supernatant was analysed using a UV spectrophotometer at a wavelength of 285–315 nm to calculate the quantity of unincorporated drug (W1) from the total amount of drug coupled with silver nanoparticle (W2).

The HAART-AgNPs percentage incorporated efficiency was calculated according to Govender et al. (Govender et al., 2006) as follows: $\%\mathrm{IE}=\frac{W_{2}-W_{1}}{W_{1}}\times 100$ = 90.52 ± 0.5%.

### 2.6 Characterization of AgNPs and HAART-AgNPs

The characterisation of AgNPs and HAART-AgNPs was previously done (Lawal et al., 2021). Briefly, Fourier Transform Infrared (FTIR) spectroscopy (Perkin-Elmer Universal ATR spectrometer, USA) was used to identify the various functional groups in the HAART + AgNPs conjugates. The ultraviolet-visible (UV-Vis) spectroscopy (Shimadzu MultSpec-1501, Shimadzu Corporation, Tokyo, Japan) was used to confirm the absorption of the conjugated HAART-AgNPs. The size and morphology of the nanoparticles were examined by a high-resolution transmission electron microscope (HR-TEM, JEOL 2100, Japan) operated at a voltage of 200 kV.

The field emission scanning electron microscope (FESEM, Carl Zeiss, Germany) operated at a voltage of 5 kV with energy dispersive X-ray (EDX, Aztec Analysis Software, England) was used to determine the elemental components.

### 2.7 Blood glucose level and metabolic activities

The weekly fasting blood glucose was determined using a portable glucometer (Sigma-Aldrich, Durban, South Africa), and the blood sample was obtained through the tail vein. The metabolic activities (characterised by calorie intake, water intake, urine volume and the number of faecal pellets) were monitored in individual rats using a novel metabolic cage.

### 2.8 Behavioural assessment

#### 2.8.1 Open field test (OFT) to measure the anxiety and explorative behaviours

On day 79 of the experiment, the animals were evaluated for spontaneous and anxiety-like behaviours using the open field test. The open field apparatus consists of a large rectangular box measuring 70 cm long × 70 cm wide × 35 cm high with several 15 cm × 15 cm squares. Animals were placed in the centre of the squares and were monitored for 5 min. The parameters for locomotion and anxiety-like behavioural activities were measured and recorded (Bădescu et al., 2016, Eilam, 2003).

### 2.9 Neurochemical analysis

#### 2.9.1 Preparation of brain homogenates

After eight weeks of treatment, all animals were anaesthetised using the excessive isoflurane inhalation method and euthanised by decapitation. The brains were harvested and immediately rinsed in cold phosphate-buffered saline (PBS). The prefrontal cortex was dissected in accordance with the Chiu procedure (Chiu et al., 2007). Then, 0.5 g of the prefrontal cortex (n = 7) was dissected on the ice tray, thawed, and homogenised in 10% phosphate buffer (0.1 M, pH 7.5). The homogenates were centrifuged for 10 mins at 20,000 g and 4 °C. The supernatants were then obtained for neurochemical analyses.

#### 2.9.2 Determination of superoxide dismutase (SOD), catalase (CAT) and malondialdehyde (MDA), and reduced glutathione level (GSH)

Prefrontal cortex tissue homogenates were used to measure the concentration of reduced glutathione (GSH), superoxide dismutase (SOD), catalase (CAT) and malondialdehyde (MDA) by spectrophotometric assay. Reduced glutathione (GSH) level was assessed using the Ellman protocol (Ellman, 1959). Superoxide dismutase (SOD) activity and catalase (CAT) were determined as reported (Aebi, 1974, Kakkar et al., 1984). Malondialdehyde (MDA) level was determined by measuring the content of thiobarbituric acid (TBA) reactive products using the method of Mkhwanazi et al. (Mkhwanazi et al., 2014).

#### 2.9.3 Analysis of inflammatory biomarkers

The concentrations of tumour necrosis factor-α (TNF-α) and interleukin (IL)− 1β were quantified in the prefrontal cortex homogenates using their specific ELISA kits (Elabscience Biotechnology Co., Ltd., Houston, TX, USA) according to the manufacturer’s instructions.

#### 2.9.4 Brain tissue processing for microscopic study

The prefrontal cortex (n = 2) was carefully removed and weighed, post-fixed in 10% neutral buffer formalin (NBF) for 1 h and transferred to 15% sucrose in phosphate-buffered saline (PBS) until they sunk (24 h). Afterwards, the tissue was transferred to 30% sucrose in PBS until they sunk and finally fixed in 10% NBF for histology and immunochemistry through paraffin embedding. The tissues were sectioned at 5 µm using Leica RM 2255 microtome, cleared in xylene, hydrated in decreasing alcohols, stained with Haematoxylin and Eosin (H&E) dye, and mounted with dibutyl phthalate poly­styrene xylene (Djidja et al., 2017).

#### 2.9.5 Immunohistochemical (IHC) analysis

The uniform random sampling of the prefrontal cortex was used for the primary antibody (anti-GFAP). The sections from the prefrontal cortex were washed in PBS (2 ×10 min) at 4 °C and pre-incubated in 0.1 M PBS, 5% normal goat serum with 0.4% Triton X-100%, and 1% bovine serum albumin for one hour at 4 °C. Then, the sections were directly incubated in the primary antibody diluted in the PBSA -Triton (PBSAT: PBS 0.01 M, PH 7.4, 0.1% of Sodium Azide and 0.3%.

Triton X 100) and prepared for 72 h at 4 °C and under agitation. After PBST washes (2 x 10 min), the sections were incubated in 0.1 M PBS containing 2% normal goat serum and biotinylated rabbit anti-goat IgG (Secondary antibody) (1:2000) for 2 h at room temperature. They were then rinsed in PBST (2 x 10 min) and incubated with the avidin-biotin complex (AB; 1:2000) for 2 h in the room, followed by several washes (1 ×10 min in PBST and 2 ×10 min in Tris buffer (0.05 M, PH 7.6)). The peroxidase activity detection was carried out with 3–3' diaminobenzidine (DAB, 0.025%), 0.5% Nickel ammonium sulphate in tris buffer (0.1 M, pH 7.6) with 0.03% hydrogen peroxide. The immunoreactive reaction was stopped by washing the sections once in 0.1 M Tris buffer (10 min) and twice in 0.1 M PBS (10 min). Sections were dehydrated in progressive ethanol baths, cleared in 2 successive xylene baths, mounted onto gelatine-coated slides and coverslipped with Eukitt.

#### 2.9.6 Quantification of immunostained astrocytes

Immunostained astrocytes counting was conducted under an optical microscope (Olympus BH2) connected via a CCD high-performance camera (COHU) to the Scion Image stereological software (Scion Corporation, version Beta 4.0.2)-equipped computer. The counting was made in 6 sections per animal along with the rostrocaudal plane of the PFC. The total number of immunoreactivity cells was presented as mean ± standard error mean (SEM).

#### 2.9.7 Ultrastructural brain tissue processing

The brain tissues were initially sectioned into1 mm^3^ pieces and post-fixed in buffered 2.5% glutaraldehyde for 12 h, washed in phosphate buffer (3 × 5 min), and transferred in 1% osmium tetroxide for 2 h. Thereafter, the tissue was washed in phosphate buffer (3 × 5 min), dehydrated in ascending grades of acetone solutions (30%, 50%, 75%, and 100%) for 5 min each, and then embedded in Durcopan (Fluka). Ultrathin Section (1 μm in thickness) of PFC were cut using an ultramicrotome (Leica Ultracut R), contrasted by uranyl acetate and lead acetate, and the prepared tissue sections were examined by transmission electron microscopy (TEM).

The TEM analysis was carried out at the Microscopy and Microanalysis Unit (MMU), the University of KwaZulu-Natal, Westville, South Africa.

### 2.10 Statistical analysis

Data were analysed and presented as mean ± SEM. The differences between means were compared using one-way analysis (ANOVA), followed by Tukey’s multiple comparison test to determine the statistical significance between the groups. All analyses were done using GraphPad Prism 8 for Windows (GraphPad Software San Diego, CA 92108). P < 0.05 was considered statistically significant.

## 3 Results

### 3.1 The characterisation of AgNPs and HAART-AgNPs

The results for the characterisation of silver nanoparticles and highly active antiretroviral therapy-silver nanoparticle conjugates were previously published (Lawal et al., 2021).

The HR-TEM investigations on the conjugated HAART-AgNPs revealed nanoparticle sizes ranging between 19 nm and 32 nm, and SEM showed that most nanoparticles are spherical in shape. The UV–vis showed an absorption peak between 315 and 320 for the nanocomposites, whereas EDX spectroscopy revealed a percentage of silver (Ag) and other elemental compositions such as oxygen, chlorine, fluorine, carbon, phosphorus, sodium, and copper. Notably, FTIR revealed the functional groups related to AgNPs and HAART, such as O-H, C-F, C-Cl, N-H and C-N (Lawal et al., 2021).

### 3.2 HAART-AgNPs reduces blood glucose level in diabetic rats

**Fig. 2:**
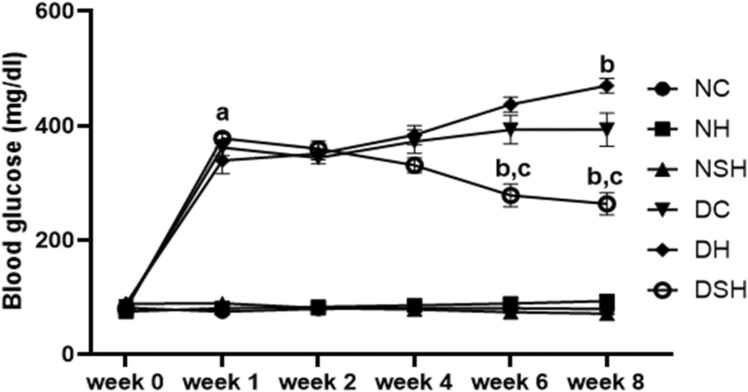
Illustrate the weekly changes in blood glucose level in non-diabetic and diabetic rats treated with either vehicle, HAART, or HAART-AgNPs. NC= nondiabetic control, NH=non-diabetic + HAART, NSH=non-diabetic+ silver nanoparticles + HAART, DC= diabetic control, DH=diabetic + HAART, DSH= diabetic +silver nanoparticles+ HAART. ^a^ vs NC; ^b^p < 0.05 v DC, ^c^p < 0.05 v DH, (n = 7).

Blood glucose levels increased significantly in all the diabetic groups (DC, DH and DSH) one-week post-STZ vs non-diabetic group (NC). Diabetic rats administered HAART (group DH) had a significant increase (p < 0.05) in blood glucose levels compared with diabetic control. In contrast, rats administered HAART-AgNPs (group DSH) had a substantial decrease in blood glucose after eight weeks of treatment vs diabetic control (DC) and diabetic treated rats only (group DH). Conversely, there was no significant difference in blood glucose levels in the non-diabetic groups (Fig. 2).

### 3.3 HAART-AgNPs improves metabolic activities

Table 1 shows the effects of HAART and HAART-AgNPs on metabolic activities and anxiety-like behaviour in non-diabetic and diabetic rats. The metabolic biomarkers significantly (water intake, urine volume, food consumption and faecal pellets) increased in the diabetic control group (DC) compared to the non-diabetic control (NC). The water intake and faecal pellets significantly increased in group DH (diabetic plus HAART) (72.13 ± 1.394) compared with the diabetic control (62.25 ± 1.88). Interestingly, both water intake and faecal pellets significantly decreased in group DSH (diabetic + HAART-AgNPs) (48.13 ± 2.10) compared to DH (72.13 ± 1.394).

**Table 1:** Metabolic activity parameters.

**Groups**	Water intake (cm^3^)	Urine Volume (cm^3^)	Food intake (mg)	Faecal pellets
**NC**	36.388 ± 0.53	19.75 ± 0.25	21.69 ± 1.23	17.38 ± 1.22
**NH**	39.13 ± 0.22	20.63 ± 1.19	23.78 ± 0.88	20.00 ± 1.19
**NSH**	36.63 ± 1.15	20.75 ± 0.31	22.96 ± 1.61	16.63 ± 1.32
**DC**	62.25 ± 1.88^**aa**^	47.00 ± 3.09^**aa**^	34.63 ± 1.99^**a**^	24.38 ± 1.10^**a**^
**DH**	72.13 ± 1.394^**b**^	58.50 ± 2.19	41.30 ± 2.47	32.25 ± 2.23^**b**^
**DSH**	48.13 ± 2.10^**c**^	39.25 ± 3.69	34.85 ± 1.72	20.38 ± 1.45^**c**^

Table 1: Effect of HAART-AgNPs on metabolic activities (water intake, urine volume, food intake, and faecal pellet number) in diabetic rats. ^a^p < 0.05, ^aa^p < 0.0001 vs NC, ^b^p < 0.05 v DC, ^c^p < 0.05 v DH. NC=nondiabetic control, NH=non-diabetic +HAART, NSH=non-diabetic+ silver nanoparticles + HAART, DC= diabetic control, DH=diabetic + HAART, DSH= diabetic +silver nanoparticles+ HAART, (n = 7).

### 3.4 The HAART-AgNPs mitigates anxiety-like behaviours in the open field test

**Fig. 3:**
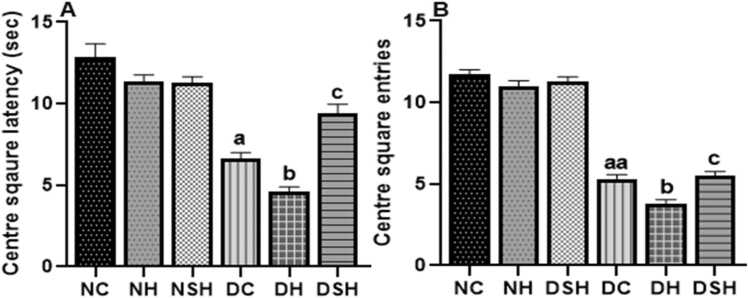
Effect of HAART-AgNPs on anxiety-like behaviour in diabetic rats. ^a^p < 0.05, ^aa^p < 0.0001 vs vs NC, ^b^p < 0.05 v DC, ^c^p < 0.05 v DH. NC= nondiabetic ^c^ontrol, NH=non-diabetic + HAART, NSH=non-diabetic+ silver nanoparticles + HAART, DC= diabetic control, DH=diabetic + HAART, DSH= diabetic + silver nanoparticles+ HAART. A= center square latency, B= center square entries, (n = 7).

Fig. 3 shows the effects of HAART and HAART-AgNPs on anxiety-like behaviours in non-diabetic and diabetic rats. There was a significant reduction (p < 0.05) in latency to leave the centre and centre square entries of group DC compared to group NC. Group DH (diabetic +HAART) had significantly reduced latency and centre square entries compared to group DC. Notably, group DSH showed a significant (p < 0.05) increase (9.375 ± 0.596) in latency compared to group DH (6.625 ± 0.3750). The centre square entries were significantly higher in group DSH (AgNPs + HAART) (5.500 ± 0267) compared with group DH (3.813 ± 0.230).

### 3.5 HAART-AgNPs increases locomotion activities

**Fig. 4:**
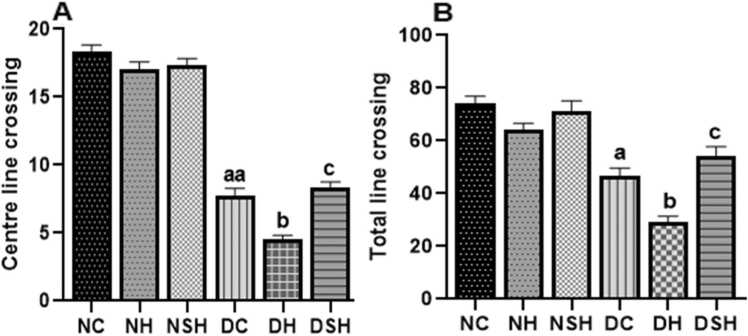
Effect of HAART-AgNPs on locomotion in diabetic rats. ^a^p < 0.05, ^aa^p < 0.0001 vs NC, ^b^p < 0.05 v DC, ^c^p < 0.05 v DH. NC= nondiabetic ^c^ontrol, NH=non-diabetic + HAART, NSH=non-diabetic+ silver nanoparticles + HAART, DC= diabetic control, DH=diabetic + HAART, DSH= diabetic +silver nanoparticles+ HAART. A= Centre line crossing, B= total line cross, (n = 7).

Fig. 4 shows the effects of HAART and HAART-AgNPs on locomotion activities in non-diabetic and diabetic rats. The indices of locomotion activities (Centre line crossing and total line crossing) significantly (p < 0.05) reduced in the DC group compared to group NC. The diabetic rats administered HAART (group DH) showed a significant (p < 0.05) reduction in locomotion compared to group DC and group DSH.

Interestingly, there was a significant increase in centre line cross in rats administered with HAART-AgNPs (group DSH) compared with group DH.

### 3.6 HAART-AgNPs reduces prefrontal cortex inflammatory biomarkers (TNF-α and IL-1β)

**Fig. 5:**
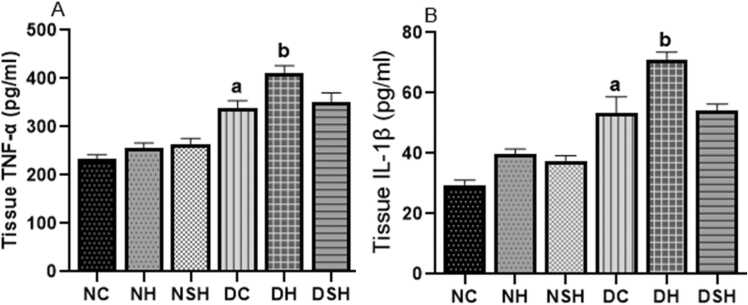
Effect of HAART-AgNPs on inflammatory response in the Prefrontal cortex of diabetic rats. ^a^p < 0.05 vs NC, ^b^p < 0.05 v DC. NC= nondiabetic control, NH=non-diabetic + HAART, NSH=non-diabetic+ silver nanoparticles + HAART, DC= diabetic control, DH=diabetic + HAART, DSH= diabetic +silver nanoparticles+ HAART. TNF-α = tumour necrosis factor- alpha, IL-1β = interleukin-1 beta, A= TNF-α, B= IL-1β, (n = 7).

Fig. 5(a-b) shows the effects of HAART and HAART-AgNPs on inflammatory markers in non-diabetic and diabetic rats. The concentration of inflammatory biomarkers (TNF-α and IL-1β) significantly (p < 0.05) increased in the DC group compared to the NC group. The diabetic rat administered HAART (group DH) showed a significant (p < 0.05) increase in both TNF-α and IL-1β compared to the DC rat. However, the diabetic rat administered HAART-AgNPs (group DSH) showed a reduction in inflammatory biomarkers compared with HAART only but not significant.

### 3.7 HAART-AgNPs enhances antioxidant enzymes activities

**Fig. 6:**
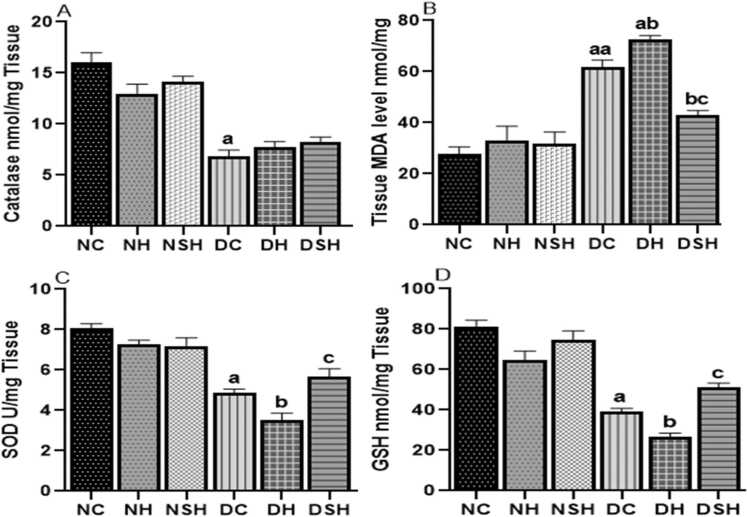
Effect of HAART on oxidative stress in the Prefrontal cortex of diabetic rats. ^a^p < 0.05 vs NC, ^b^p < 0.05 v DC, ^c^p < 0.05 v DH. NC= nondiabetic control, NH=non-diabetic + HAART, NSH=non-diabetic+ silver nanoparticles + HAART, DC= diabetic control, DH=diabetic + HAART, DSH= diabetic +silver nanoparticles+ HAART. A= Catalase, B= Malondialdehyde, C=superoxide dismutase (SOD), D= reduced glutathione (GSH), (n = 7).

Fig. 6(a-d) shows the effect of HAART and HAART-AgNPs on oxidative stress biomarkers. The diabetic control group had a significant (p < 0.05) increase in MDA level and a significant (p < 0.05) decrease in catalase, SOD and GSH compared with the non-diabetic control group.

Administration of HAART to the diabetic rat (DH) significantly increased MDA with a reduction in SOD and GSH compared to diabetic control. However, administration of HAART-AgNPs to diabetic animals (DSH) caused a significant decrease in MDA levels and increased GSH and SOD levels compared with group DH (p < 0.05).

### 3.8 HAART-AgNPs protects GFAP-positive astrocytes in the prefrontal cortex

**Fig. 7:**
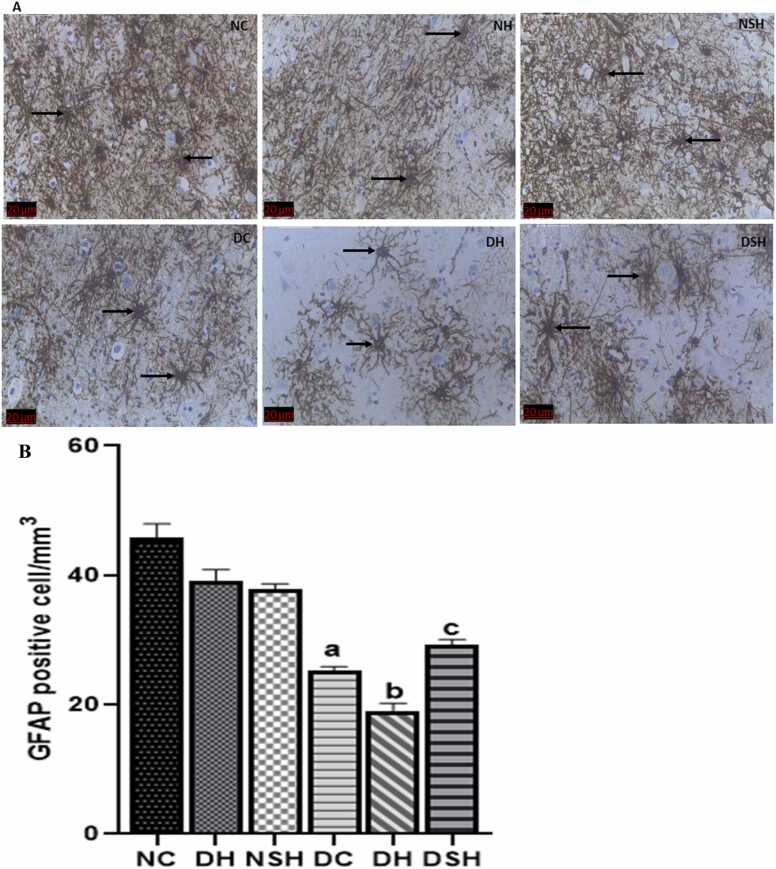
A Prefrontal cortex GFAP-positive astrocytes in diabetic and non-diabetic groups, NC= nondiabetic control, NH=non-diabetic + HAART, NSH=non-diabetic+ silver nanoparticles + HAART, DC= diabetic control, DH=diabetic + HAART, DSH= diabetic +silver nanoparticles+ HAART. Black arrow indicates GFAP-positive astrocyte.Fig. 7B Effect of HAART-AgNPs on GFAP positive astrocytes in the Prefrontal cortex. NC= nondiabetic control, NH=non-diabetic + HAART, NSH=non-diabetic+ silver nanoparticles + HAART, DC= diabetic control, DH=diabetic + HAART, DSH= diabetic +silver nanoparticles+ HAART, GFAP= glial fibrillary acidic protein. ^a^p < 0.05 v NC, ^b^p < 0.05 v DC, ^c^p < 0.05 v DH. Black arrow indicates immunostained astrocytes, (n = 2).

Fig. 7 A and B show the effect of HAART and HAART-AgNPs on GFAP-positive astrocytes. There was a significant reduction in GFAP positive astrocytes in the prefrontal cortex of diabetic control (DC) compared to non-diabetic control (NC). Administration of HAART to diabetic rats (DH) caused a significant reduction in GFAP-positive astrocytes compared with group DC. However, HAART-AgNPs administration to diabetic rats (DSH) significantly increased GFAP-positive astrocytes compared to group DH.

### 3.9 HAART-AgNPs prevents prefrontal cortex neuronal injury

**Fig. 8:**
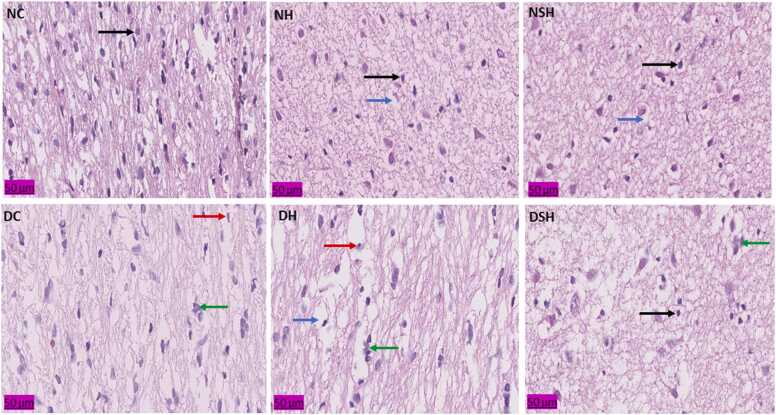
Effect of HAART-AgNPs on Prefrontal cortex neuronal cell. NC= nondiabetic control, NH=non-diabetic + HAART, NSH=non-diabetic+ silver nanoparticles + HAART, DC= diabetic control, DH=diabetic + HAART, DSH= diabetic +silver nanoparticles+ HAART. Black arrow = normal neuronal cell, red arrow = neuronal atrophy, green arrow= neuronal hypertrophy and blue arrow= cytoplasmic shrinkage (n = 2).

The effect of HAART and HAART-AgNPs on prefrontal cortex neuronal cells is shown in Fig. 8. The diabetic control group (DC) showed shrinkage of cytoplasm and hypertrophy of neuronal cells compared with the normal control (NC). The non-diabetic groups (NH and NSH) administered with HAART and HAART-AgNPs showed shrinkage of cytoplasm. Notably, administration of HAART-AgNPs to diabetic rats (group DSH) showed more normal neuronal cells with few neuronal hypertrophies compared with diabetic rats administered with HAART only (group DH).

### 3.10 HAART-AgNPs protects ultrastructural organelles of the prefrontal cortex

**Fig. 9:**
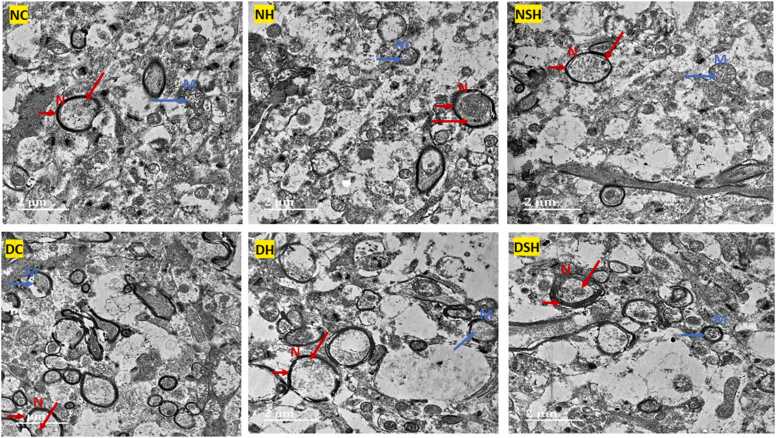
Effect of HAART-AgNPs on Prefrontal cortex neuronal organelles. NC= nondiabetic control, NH=non-diabetic + HAART, NSH=non-diabetic+ silver nanoparticles + HAART, DC= diabetic control, DH=diabetic + HAART, DSH= diabetic +silver nanoparticles+ HAART. N indicates Nucleus (short red arrow = nuclear membrane, long red arrow= nucleoli), M indicates Mitochondria (blue arrow= mitochondrial cristae) (n = 2).

Fig. 9 shows the effect of HAART-AgNPs on prefrontal cortex neuronal organelles. The control group (NC) showed a normal nucleus (N) with a double-layered nuclear membrane and the presence of nucleoli. The mitochondria in the control group showed intact membrane and the presence of cristae within the mitochondria. All treated groups presented with a various nucleus and mitochondrial alterations. The diabetic group (DC) and the diabetic group treated with HAART showed ruptured and vacuolated mitochondria (M) with degenerated nucleoli. However, the diabetic rats (group DSH) treated with HAART-AgNPs showed an improved double-layered membrane and presence of nucleoli and mitochondrial cristae compared with diabetic rats treated with HAART only.

## 4 Discussion

This study examined the effect of HAART-silver nanoparticles conjugate on metabolic, behavioural, molecular, histological, and ultrastructural changes associated with prolonged administration of HAART in diabetic rats.

HAART is required at higher doses for a lifetime to maintain an undetectable viral load in people living with HIV, which predisposes them to systemic toxicity and metabolic disorders such as diabetes mellitus (Nduka et al., 2017, Ergin et al., 2020). Recently, silver nanoparticles have been used for a wide range of applications in the biomedical field, such as antiviral, antioxidant and antidiabetic agents, due to their unique properties and possibly the synergistic effects on therapeutic agents (Vadlapudi and Amanchy, 2017, Essghaier et al., 2022). Thus, conjugation of AgNPs to HAART may exert significant benefits, such as reducing the dosage of HAART needed for therapy and inhibiting some pathophysiological mechanisms mediated by HAART in contribution to diabetes (Lawal et al., 2021, Olojede et al., 2022). Although, the issues related to the toxicity profile of AgNPs on the biological tissues have been raised (Fadaka et al., 2022). Conversely, studies have reported that the toxicity profile of AgNPs depends on several factors such as nanoparticles size, shape, morphology, dimension, and capping agent (Alkaladi et al., 2014, Fadaka et al., 2022).

In the current study, the characterisation of HAART loaded silver nanoparticles with various concentrations (0.5 M, 1 M, 1.5 M, and 2 M) showed nanoparticles sizes from 19 nm to 32 nm and a spherical shape. Interestingly, previous studies have reported that nanoparticles with small-medium particle size and spherical shape are sensitive and less toxic to the biological tissues (Lara et al., 2010, Lee et al., 2013, Agnihotri et al., 2014, Ferdous and Nemmar, 2020). In addition, a study has confirmed that there are no adverse effects of AgNPs of 30 nm with spherical shape on the alveolar epithelial cells (Stoehr et al., 2011). Therefore, we hypothesised that HAART loaded silver nanoparticles may improve therapeutic efficacy and reduce neurological disorders associated with high dosage and long-term exposure to HAART.

In this study, the diabetic rats treated with HAART had persistently increased blood glucose levels across the weeks. This result shows that the chronic administration of HAART contributed to the hyperglycaemic effect of diabetes. The development of hyperglycaemia has been reported with the prolonged use of HAART in people living with HIV (Sharma, 2014), which has been attributed to insulin resistance, mitochondrial dysfunction, and metabolic disorders (Avari and Devendra, 2017). Conversely, a significant decrease in blood glucose was observed in rats treated with HAART-silver nanoparticles conjugate (HAART-AgNPs) compared with HAART-treated rats only. This glycaemic control may be due to the potential of silver nanoparticles to increase insulin secretion via insulin-like growth factor-I, thereby promoting glucose uptake (Olojede et al., 2022). A similar study has shown the antidiabetic activity of silver nanoparticles via up-regulation of insulin receptors and higher expression of glucokinase genes (Alkaladi et al., 2014).

The metabolic disturbances of HAART were observed as evidenced in increased defecation and water intake of diabetic rats. Literature has shown that an increase in defecation is a valuable indicator of anxiety-like behaviours and is frequently observed in highly emotional animals (Hall, 1934, Crumeyrolle-Arias et al., 2014). In this study, the administration of HAART to the diabetic rats exacerbates anxiety-like behaviour compared to diabetic control and diabetic rats treated with HAART-AgNPs. Evidence of behavioural deficits and anxiogenic effects of HAART was seen in the open field test. The centre square entries, the latency to leave the centre square, the centre line cross and the total line cross were significantly reduced in the diabetic HAART-treated group. Despite the benefits of the backbones of HAART components (NRTIs and NNRTIs), they are associated with neuropsychological disturbances, fatigue, and dizziness (Romao et al., 2011). More so, chronic treatment of Efavirenz (NNRTIs) has been reported to induce an anxiety-like effect in animals and humans (Raines et al., 2005, Romao et al., 2011, Cavalcante et al., 2017).

The neurological observation in the HAART-treated rats was associated with a significant increase in the prefrontal cortex MDA level. This observation may be due to the excessive production of ROS, which occurs during the intracellular phosphorylation of NRTIs in the prefrontal cortex (Schank et al., 2021). The excessive ROS production and the reduced antioxidant enzymes CAT, SOD and GSH may promote oxidative stress resulting in tissue injury. A previous study has reported that oxidative injury promotes lipid peroxidation that compromises mitochondrial biogenesis, which has been implicated in HAART-induced mitochondrial dysfunction (Schank et al., 2021).

In this study, HAART-AgNPs alleviates the anxiety-like behaviours in the diabetic rats via improved metabolic disturbances and anxiogenic parameters in the open field, which correlated with reduced MDA and improvement in GSH, CAT and SOD. This suggests that silver nanoparticles may alleviate the anxiogenic effects of long-term administration of HAART via their antioxidant properties by reducing ROS production during the intracellular phosphorylation of HAART. This result agrees with previous findings that reported a significant antioxidant activity of silver nanoparticles (Keshari et al., 2020). In addition, an increase in antioxidant activity has been demonstrated to improve brain cell oxidative injury and cognitive functions (Franzoni et al., 2021).

Increased oxidative stress triggers the release of pro-inflammatory cytokines (TNF-α and IL-1β), as seen in the PFC of HAART-treated and diabetic control rats. The brain tissues are particularly susceptible to oxidative stress and neuronal damage due to their low antioxidant defence system, high amount of unsaturated fatty acid, and high oxygen consumption (Salim, 2017). In this recent study, an increase in the concentration of pro-inflammatory cytokines (TNF-α and IL-1β), inhibition of antioxidants enzymes (CAT, SOD) and GSH were associated with anxiety-like behaviour in diabetic rats treated with HAART. A similar report has established an increase in TNF-α and IL-1β were associated with mood and anxiety disorders (Quagliato and Nardi, 2018). Furthermore, literature has shown that HIV patients receiving HAART are susceptible to type 2 diabetes mellitus and its neuropathic complications due to increased ROS production that promotes cellular toxicity and neuroinflammation (Sharma, 2014, Nsonwu-Anyanwu et al., 2017, Pang et al., 2020;).

Conversely, there was a slight decrease in the PFC concentration of pro-inflammatory cytokines (TNF-α and IL-1β) in HAART-AgNPs treated rats compared with HAART treated only (although not significant). This result suggests that silver nanoparticles may mitigate the neurotoxic effect of long-term use of HAART due to their anti-inflammatory properties. This is in line with previous studies showing the anti-inflammatory and neuroprotective effects of silver nanoparticles (Seethalakshmi, 2015, Tyavambiza et al., 2021).

Several studies have reported the role of PFC and astrocytic cells in anxiety disorders (Sofroniew and Vinters, 2010, Tovote et al., 2015, Hare and Duman, 2020). HAART caused astrocyte dysfunction and a decrease in PFC GFAP-positive astrocyte number in the diabetic rats. The dysfunction and decrease in astrocytes may be attributed to the neurological deficits observed in diabetic rats treated with HAART.

In this study, an increase in the PFC GFAP-positive cells was observed in HAART-AgNPs treated animals. Silver nanoparticles offer an advantage for delivering therapeutic agents due to their unique physicochemical characteristics, antioxidant, and anti-inflammatory properties (Vadlapudi and Amanchy, 2017). The improvement observed in PFC GFAP-positive cells may be attributed to the antioxidant and anti-inflammatory properties of silver nanoparticles to delay or prevent the loss of astrocytes in the PFC (Burdusel et al., 2018).

Furthermore, the administration of HAART-AgNPs to diabetic rats protects neuronal cells against oxidative injury exacerbated by HAART and diabetes. The previous study has shown the therapeutic potential of silver nanoparticles in tissue restoration and regeneration (Burdusel et al., 2018).

The mechanism by which HAART exerts its neurotoxic effects has been linked with mitochondrial damage and neuronal injury (Gnanasekaran, 2020). The evidence was seen in non-diabetic and diabetic rats treated with HAART that presented with ruptured, vacuolated mitochondria and degenerated nucleoli. However, HAART-AgNPs alleviates the anxiety-like behaviour by protecting the neuronal ultrastructural organelles (nucleus and mitochondria) in the PFC via its intrinsic anti-inflammatory and tissue restoration properties (Burdusel et al., 2018).

While the literature has reported the neurotoxic effects of silver nanoparticles (Węsierska et al., 2018, Greish et al., 2019b), there is substantial evidence that the neurotoxic effects of silver nanoparticles depend on various factors, particularly the synthesis method (Alkaladi et al., 2014, Seethalakshmi, 2015). The reduction of Ag^+^ to Ag^0^ using a 1.5 M trisodium citrate (TSC) concentration as a reducing and stabilising agent with a nanoparticle size between 19 and 35 nm and spherical morphology may be an essential factor determining their neurotoxic effect on the PFC (Lara et al., 2010). The previous investigation of silver nanoparticles synthesis where the silver ion has been reduced to a ground state from Ag^+^ to Ag^0^_,_ and synthesised nanoparticles were within the small-medium nano-sized particle (20–50 nm) reported non-toxic to the biological tissues (Lara et al., 2010, Van Dong et al., 2012, Iravani et al., 2014). Furthermore, a study has reported that the cytotoxic effect observed in the use of silver nanoparticles is due to silver ions exposure (Wang et al., 2014). Therefore, the reduction of Ag^+^ to Ag^0^ using a 1.5 M trisodium citrate (TSC) concentration as a reducing and stabilising agent with a nanoparticle size between 19 and 35 nm and spherical morphology may be an essential factor in determining that reduced neurotoxic effects and improved antioxidant function of nanoparticles in our research.

## 5 Conclusion

Data from this study showed that the administration of HAART aggravates anxiety-like behaviours and promotes neurotoxic effects on the PFC of diabetic rats. However, HAART conjugated with silver nanoparticles mitigates the anxiogenic effects of HAART, preserves PFC GFAP-positive cells and ultrastructural neuronal organelles, and reduces neuronal damage by reducing oxidative injury and inflammatory damage. The conjugation of silver nanoparticles and HAART as a treatment regimen in HIV may be explored to enhance drug delivery while reducing the risk of neurological disorders (e.g., anxiety) associated with prolonged use of HAART.

## Ethical statement

All animals were handled according to the National Institute of Health Guide for the Care, and Use of Laboratory Animals (NIH Publications No. 80-23), revised in 1996. The animal laboratory procedures were approved by the Animal Ethics Committee of the University of KwaZulu-Natal (AREC/044/019D).

## Consent for publication

All authors have read and accepted responsibility for the content of the manuscript.

## Funding

This work was supported by the College of Health Sciences, University of KwaZulu-Natal, South Africa (grant number 640967).

## CRediT authorship contribution statement

**Sodiq Kolawole Lawal:** Conceptualisation, Methodology, Investigation, Data curation, Formal analysis, Writing-original draft, Funding acquisition. **Samuel Oluwaseun Olojede:** Conceptualisation, Methodology, Investigation. **Ayobami Dare:** Methodology, Validation, Investigation, Formal analysis, Writing – original draft. **Oluwaseun Samuel Faborode:** Formal analysis, Validation, Visualisation, Writing – review & editing. **Sheu Oluwadare Sulaiman:** Formal analysis, Writing – review & editing. **Edwin Coleridge Naidu:** Resources and Visualisation, Writing – review & editing, Supervision. **Carmen Olivia Rennie:** Resources, Supervision, Project administration, Writing – review & editing, Funding acquisition. **Onyemaechi Okpara Azu:** Conceptualisation, Writing – review & editing.

## Conflict of interest

The authors have no conflict of interest to declare.
